# Analyzing artificial intelligence systems for the prediction of atrial fibrillation from sinus-rhythm ECGs including demographics and feature visualization

**DOI:** 10.1038/s41598-021-02179-1

**Published:** 2021-11-23

**Authors:** Pietro Melzi, Ruben Tolosana, Alberto Cecconi, Ancor Sanz-Garcia, Guillermo J. Ortega, Luis Jesus Jimenez-Borreguero, Ruben Vera-Rodriguez

**Affiliations:** 1 Biometrics and Data Pattern Analytics Lab, Escuela Politecnica Superior, Universidad Autonoma de Madrid, Calle Francisco Tomas Y Valiente, 11, C-235, 28049 Madrid, Spain; 2 Instituto de Investigacion Sanitaria del Hospital Universitario de La Princesa, Madrid, Spain; 3 Science and Technology Department, National University of Quilmes, Bernal, Argentina; 4 Consejo Nacional de Investigaciones Cientificas y Tecnicas, CONICET, Buenos Aires, Argentina; 5 CIBERCV, Centro de Investigacion Biomedica en Red Enfermedades Cardiovasculares, Madrid, Spain

**Keywords:** Atrial fibrillation, Machine learning

## Abstract

Atrial fibrillation (AF) is an abnormal heart rhythm, asymptomatic in many cases, that causes several health problems and mortality in population. This retrospective study evaluates the ability of different AI-based models to predict future episodes of AF from electrocardiograms (ECGs) recorded during normal sinus rhythm. Patients are divided into two classes according to AF occurrence or sinus rhythm permanence along their several ECGs registry. In the constrained scenario of balancing the age distributions between classes, our best AI model predicts future episodes of AF with area under the curve (AUC) 0.79 (0.72–0.86). Multiple scenarios and age-sex-specific groups of patients are considered, achieving best performance of prediction for males older than 70 years. These results point out the importance of considering different demographic groups in the analysis of AF prediction, showing considerable performance gaps among them. In addition to the demographic analysis, we apply feature visualization techniques to identify the most important portions of the ECG signals in the task of AF prediction, improving this way the interpretability and understanding of the AI models. These results and the simplicity of recording ECGs during check-ups add feasibility to clinical applications of AI-based models.

## Introduction

Atrial fibrillation (AF) is one of the most common sustained arrhythmias, increasing the risk of strokes^1^, heart failure^2^, and other heart-related complications^3^. AF is often asymptomatic and it may remain undiagnosed until a first manifestation of stroke^4,5^. Approximately 20% of patients who have a stroke associated to AF are first diagnosed with AF at the time of stroke or shortly thereafter^6^. The identification of those possible individuals at risk of developing AF, and their consequent treatment, can reduce mortality and strokes as well as health care costs^7^.

Despite this, a commonly accepted standardized assessment of symptoms is missing, partly because of the complex clinical decision-making process in patients with AF^8^. The electrocardiogram (ECG) provides meaningful information that may contribute substantially to the detection of present AF and estimation of future AF risk^9^, even if existing commercial ECG interpreters show limitations and errors^10,11^. Traditional techniques for AF prediction combine discrete features extracted from ECGs and machine learning classifiers^12,13^. Promising features for the prediction of AF, such as P wave duration, RR interval duration, and PQ segment level, have been considered in the literature^14^. However, this kind of approaches takes into account only little information from the ECG and require a time-consuming phase to extract and select handcrafted features^15^, also prone to errors.

In the last decade, artificial intelligence (AI) has brought a considerable breakthrough in many fields such as image and signal processing^16,17^. Important advances have also occurred with application to ECGs. Deep neural networks (DNNs) can automatically learn representative and robust features from ECG signals to replace or complement traditional handcrafted features^18,19^. The automatic classification of ECGs according to different heart rhythms has already achieved important results^20^. Also, DNNs have shown to be able to identify in 12-lead ECGs subtle abnormalities due to structural derangements^21^. Atrial structural changes may occur before AF. In particular, structural remodeling is seen as the main contributor for initiation and persistence of AF^22^.

The use of DNNs to process ECGs with normal sinus rhythm and assess the risk of developing future AF has been investigated in previous studies achieving good results^23–25^. However, in these previous studies there is a lack of analysis and comparison of different AI approaches^23,24^. Also, the prevalence of AF in adults is significantly related to age, while its relationship with sex is mostly unclear^26–28^. In addition, the details of the study population are usually described summarily in previous articles in the literature, without making any distinction between healthy and AF suffering patients. Instead, we believe it is important to point out the differences between the two classes of patients^29^ and to take them into consideration when evaluating the results of the study. Finally, in previous articles there is a missing interpretability and explainability of the AI models. Despite DNNs have outperformed traditional techniques, they continue to be treated as black-box tools which map given inputs to classification outputs. Incorporating DNNs into medical diagnosis, planning, and control requires a higher level of trust of the machine capabilities^30^ and the possibility to explain the processes carried out by DNNs.

In this study we take all these aspects into consideration. We carry out a comparative analysis of different AI models, some of them based on DNNs, to identify subtle traits that may anticipate pending episodes of AF in ECGs with normal sinus rhythm. The AI models may also reveal undiagnosed AF in some patients. We consider a database of 12-lead ECGs collected from a large cohort of 122,394 patients at La Princesa University Hospital (Madrid, Spain).

Studies in the literature agree on the increase of incidence in elderly persons but suggest diverse evidences regarding its relationship with sex^26–28^. In this study we compare multiple scenarios, featured with different age distributions between healthy and AF suffering patients. We also compare the performance achieved by AI models in predicting AF across multiple age-sex-specific group of patients to observe if the model performance is affected by age and sex of patients.

Finally, we try to overcome the limitation of interpretability by applying AI techniques for feature visualization to the processed ECG samples. We discover which portions of ECGs are the most exploited for AF prediction and draw a comparison of the contributions in AF prediction provided by each lead.

In all the experiments, we keep in mind that ECGs collected too early may not even contain a subtle notice of AF while ECGs collected in the days before AF may refer to hospitalized patients who also suffered other diseases or underwent surgery. If the latter, AF is induced by causes that are out of the scope of this work, such as systemic inflammation, and it is important to properly handle this aspect.

The use of AI-enhanced techniques for predicting AF from ECGs recorded during sinus rhythm is also hindered by the inadequate evidence that screening with ECG is more effective than usual care^6^. Potential harms of finding AF earlier in people with no symptoms include unnecessary treatment or overtreatment, which can have major side effects such as bleeding caused by blood thinners or complications from more invasive heart procedures^31^.

The goal of this study, along with the early detection of AF by processing ECG samples recorded during normal sinus rhythm, is the analysis of those aspects that affect AF prediction and have not been investigated enough in previous studies. Such analysis leads to the implementation of more reliable and trusted AI-enhanced predictors of AF. The properties of ECGs make them very convenient for the task: they can be quickly recorded in a doctor’s office or by means of wearable devices and contain ready-to-use information suitable for AF prediction.

## Results

### Study population

The study is conducted with a database of ECGs collected from a large cohort of 122,394 patients at La Princesa University Hospital (Madrid, Spain) between May 5, 2010, and February 4, 2019. ECGs have been recorded and processed by the Philips 12-Lead Algorithm (https://philips.to/36CPabZ) that provides the raw ECG time measures along with an automatic interpretation of rhythm and extracts discrete variables related to global and lead-specific attributes of the signal. We divide patients into two groups: (i) the ones who developed AF at least once along their clinical history, and (ii) the ones who only have ECGs with interpretation of “sinus rhythm” (SR) along their clinical history. For patients in the first group, we define the AF index date as the date in which the first episode of AF occurred. For patients in the second group, we define the SR index date as the date of their last recorded ECG. In both groups, we only consider ECGs that present an automatic interpretation of “sinus rhythm”, according to our goal of predicting AF from normal-rhythm ECGs that do not contain clear evidence of AF yet. Details on the database and data cleaning operations are provided in “Methods” and Supplementary Figure S1.

These operations result in two groups of data composed of: (i) 12,198 ECGs recorded from 3,761 patients (AF group) and (ii) 64,829 ECGs recorded from 22,896 patients (SR group). Patients in AF group present a median age of 80 years (IQRs 70–86) at their AF index date and 49.61% of them are females. Patients in SR group present a median age of 64 years (IQRs 50–77) at their SR index date and 52.96% of them are females. For each patient and according to their AF or SR index date, we consider temporal intervals containing the ECGs of interest in our experiments. We define these temporal intervals “time windows” and detail them in “Methods”.

### Description of experiments

For the analysis of AI-based systems that predict AF from ECGs recorded during sinus rhythm, we divide our experiments into two main phases. The first one examines state-of-the-art AI models and different age distributions in the datasets, while the second one investigates the behavior of AI models for age-sex-specific groups of patients. In the first phase, we define six different AI models whose description is provided in “Methods”. We investigate and compare their ability to predict AF exploiting different kinds of features extracted from 12-lead raw ECGs. For each ECG sample, our AI models compute an output that represents the risk of future AF episodes (AF score). We train and evaluate the six models always keeping the same experimental protocol, to obtain a fair comparison of the performance and features exploited for prediction. The test set contains multiple ECGs for several patients. We consider multiple rules to aggregate AF scores related to the same patients, to provide a unique score representing the risk of developing AF for patients. We assess the performance of single models. Then, we average the AF scores provided by the six models for each ECG sample and observe if we can achieve any improvement in performance. We also analyze the performance of three of our models when we train and evaluate them with datasets provided with specific characteristics related to the distribution of patients age. This analysis allows to compare the impact of age in different models.

For the second phase of the analysis, we balance the datasets by matching the age distribution of patients between the AF and SR groups. We train one of our six models and evaluate its performance with multiple test sets, which represent age-sex-specific groups of patients. We establish five consecutive age ranges according to the age distribution of our data and the significant intervals provided by other studies^32,33^ to group patients into different sets: patients younger than 60, 60–70, 70–80, 80–90, and older than 90 years. For each age range we build two distinct sets of patients, one for males and one for females, and we obtain a total of ten age-sex-specific groups of patients.

Finally, we apply AI techniques for feature visualization to the ECG samples included in our test sets. Thereby we can locate on the ECG signals the most significative portions for our model to predict the future presence or absence of AF. With the information provided by feature visualization, we can quantify the contribution of each lead for AF prediction and observe the differences among age-sex-specific groups of patients.

### First phase: analysis of state-of-the-art AI models and age distributions

We build datasets for the training and evaluation of our six AI models by randomly sampling patients and ECGs in terms of age and sex. In the training set we include 5,337 ECGs from the AF group, with a maximum of five ECGs per patient. All the ECGs are recorded before the first event of AF. For validation and test sets we consider a smaller time window that contains ECGs collected between 2 months and 1 week before the first AF. In the validation set we include 85 ECGs, with a maximum of five ECGs per patient, while in the test set we include all the ECGs available in the specified time window, from a set of 60 patients randomly sampled. SR ECGs in the training and validation sets for the AF group present a median age of 79 years (IQRs 70–84) and 77 years (IQRs 72–87), respectively. In the test set, the median age of AF patients is 82.5 years (IQRs 73–88). We specify the median age of patients and not ECGs because, for each patient in the test set, we aggregate the AF scores related to their ECGs and provide a unique score representing their own risk of developing AF.

In both the training and validation sets, we select ECGs from the SR group until we obtain the same number of ECGs representing the two groups of patients. Given the higher number of patients available in the SR group, we consider at most one ECG for each SR patient. With such constraint, we aim at increasing the amount of information exploitable by our AI model. In the test set we include all the available ECGs from a set of 300 patients. SR ECGs in the training and validation sets present a median age of 63 years (IQRs 50–75) and 66 years (IQRs 54–77), respectively. In the test set, the median age of SR patients is 64 years (IQRs 50–75). This is similar to the experimental protocols considered in previous studies^9,23^ and real scenarios, where AF affects mostly older people.

We define two rules to aggregate AF scores that belong to the same patients: for each patient, we consider *i)* the average and *ii)* the maximum of their AF scores. In Table 1 we provide the results achieved with the different AI models. AUC is computed in three different ways for each model: according to the aggregated AF scores obtained with the two different rules (Avg ECGs, Max ECGs) and considering the entire amount of AF scores, without applying any aggregation rule (All ECGs). In the case of MLR, and consequently in the fusion of the six models, we discard from the test set 6 AF patients and 73 SR patients because of missing values contained in the discrete features of their ECGs. For every model, the highest AUC is obtained with the AF score aggregation by average. Hence, in the following experiments we only provide results computed with aggregation by average. The first three models, with the bottom layers of ResNet, are better than recurrent neural network (RNN) and wavelet scattering. They achieve a performance similar to multivariate logistic regressor (MLR), with the best AUC of 0.89 (0.83–0.95) obtained for ResNet with age/sex model and aggregation by average rule.

**Table 1:** Comparison of AUC results achieved for different models, according to different aggregation rules.

Model	All ECGs–AUC	Avg ECGs–AUC	Max ECGs–AUC
Resnet	0.84 (0.78–0.90)	0.87 (0.81–0.93)	0.84 (0.78–0.91)
Resnet with age/sex	0.85 (0.80–0.91)	0.89 (0.83–0.95)	0.87 (0.81–0.93)
Resnet + Recurrent layers	0.83 (0.78–0.89)	0.87 (0.81–0.93)	0.84 (0.78–0.91)
RNN	0.77 (0.70–0.83)	0.81 (0.74–0.88)	0.75 (0.68–0.83)
Wavelet Scattering	0.78 (0.71–0.84)	0.80 (0.73–0.87)	0.77 (0.69–0.84)
MLR	0.85 (0.79–0.91)	0.86 (0.80–0.93)	0.85 (0.78–0.92)
Fusion	0.85 (0.79–0.91)	0.87 (0.81–0.94)	0.86 (0.79–0.92)

The performance of six different models is evaluated with AUC. AUC for the Fusion model is computed by averaging for each ECG sample the six AF scores computed by the different models. The same data have been used to train and evaluate the six models. The only exception refers to MLR and Fusion models, where ECG samples with missing discrete values have been discarded from the analysis. AUC results are computed according to three different aggregation rules: *i)* no aggregation (All ECGs), *ii)* average of AF scores related to the same patient (Avg ECGs), and *iii)* selection of the highest AF score for each patient (Max ECGs). 95% confidence intervals are reported in brackets.

*ECG* Electrocardiogram, *AUC* Area Under the Curve, *RNN* Recurrent Neural Network, *MLR* Multivariate Logistic Regression.

At this point, we focus on three models (ResNet with age/sex, ResNet, and MLR) and evaluate the performance achievable by training and evaluating them with different datasets. We keep the same AF data, and we randomly sample new SR data complying with the constraints defined for the previous selection of SR data. We consider two new scenarios: (i) SR data sampling to balance the age distributions of AF patients (median ages: 75 years (IQRs 67–80) in training, 77 years (IQRs 72–87) in validation and 82.5 years (IQRs 73–88) in test sets) and (ii) SR data sampling of patients younger in average than all the previously considered sets of data (median ages: 53 years (IQRs 43–60) in training, 53 years (IQRs 45–59) in validation and 53 years (IQRs 43.375–60) in test sets). In Table 2 we provide the AUC results achieved for different datasets and AI models.

**Table 2:** Comparison of AUC results achieved for different models, according to different scenarios.

Model	Random sampling	Balancing distributions	Young SR data
Resnet	0.87 (0.81–0.93)	0.77 (0.70–0.84)	0.92 (0.87–0.97)
Resnet with age/sex	0.89 (0.83–0.95)	0.79 (0.72–0.86)	0.98 (0.95–1.00)
MLR	0.86 (0.80–0.93)	0.71 (0.64–0.78)	0.97 (0.94–1.00)

In each scenario (random sampling, balancing distribution, young SR data) the same ECG samples have been used to train and evaluate the three different AI models. The only exception refers to MLR, where ECG samples with missing discrete values have been discarded from the analysis. AUC results are computed according to the aggregation rule by average. 95% confidence intervals are reported in brackets.

*ECG* Electrocardiogram, *MLR* Multivariate Logistic Regression.

We observe that by increasing the difference between age distributions, we can also increase the performance of the models (AUC = 0.79 with balanced age distributions, AUC = 0.89 with random sampling, and AUC = 0.98 with younger SR data). Related studies should always address this aspect and detail any difference in the age distributions of patients belonging to the two opposite groups, this has not been considered in most of previous studies^23–25^. It is very simple to boost the model performance by selecting opportune sets of patients. ResNet with age/sex always achieves the highest AUC. It is important to notice that the ResNet model performs better than MLR in terms of AUC only in the scenario with balanced age distributions, where features other than age increase their significance and may be better exploited by complex models. The MLR system is highly dependent on the age distributions. In fact, for the dataset with young SR patients, MLR even almost outperforms ResNet with age/sex. In Table 3 we provide additional metrics related to the three models, computed in the scenario of balancing age distributions.

**Table 3:** Comparison of the performances obtained for different models, in the scenario of balancing distributions.

	AUC	Sensitivity	Specificity	DOR	F_1_ score
Resnet	0.77 (0.70–0.84)	65.00% (51.60–76.87)	70.67% (65.16–75.76)	4.47 (2.49–8.04)	0.42 (0.30–0.45)
Resnet with age/sex	0.79 (0.72–0.86)	78.33% (65.80–87.93)	69.33% (63.78–74.50)	8.17 (4.22–15.84)	0.47 (0.35–0.50)
MLR	0.71 (0.64–0.78)	66.67% (52.53–78.91)	67.32% (60.44–73.69)	4.12 (2.18–7.79)	0.46 (0.36–0.54)

In the scenario of balancing distributions, we compare the three models considered. To compute the metrics additional to AUC, we set a threshold for each model, as described in “Methods”. Performances are assessed according to the aggregation rule by average. 95% confidence intervals are reported in brackets.

*M* Male, *F* Female, *AUC* Area Under the Curve, *DOC* Diagnostic Odd Ratio.

We also identify an interesting pattern by analyzing the age of misclassified patients in the scenario of balanced age distributions. Even if the mean age of patients in both AF and SR groups is equal (79 years), AF patients classified as SR patients are younger in average (74.43 years) and SR patients classified as AF patients are older in average (82.42 years). We consider for this analysis the output computed by the ResNet model, as it does not require the age of patients as input parameter.

### Second phase: analysis of age-sex-specific groups of patients

For the second phase of the analysis, we build new datasets with the double aim of: (i) balancing the age distribution between AF and SR groups of patients and (ii) equally representing all the age-sex-specific groups of patients under investigation. We train the ResNet model, which does not require the age and sex of patients as input parameters, to evaluate the performance of age-sex-specific groups of patients. With ResNet, we can also apply AI techniques for the visualization of features learned by the model in a straightforward way^34^. We include in the training set 400 SR ECGs from AF patients and 400 SR ECGs from SR patients for each age-sex-specific group of patients, with a maximum of five ECGs per patient, in compliance with the time windows specified in “Methods”. In cases with not enough samples, we also include in the training set SR ECGs from AF patients recorded after their AF index date and SR ECGs from SR patients recorded between one and two years before their SR index date. The age-sex-specific groups with patients above 90 years and the one with female patients between 18 and 59 years result less represented than the other groups of patients due to lack of data. In conclusion, the AI model is trained with 6,382 ECGs (around 4,500 patients) and evaluated on ten different test sets, representing the ten different age-sex-specific groups of patients. The age distribution between AF and SR data is balanced in all the datasets considered, as detailed in Supplementary Table S1. The experimental results are provided in Table 4. The ResNet model predicts AF in the different age-sex-specific groups of patients with different precisions. For male patients, the model is more accurate in the prediction of AF for older patients. Regarding the two youngest age ranges, the model performance is higher for female patients than for male patients. It is the opposite between male and female patients in the age-sex-specific groups that refer to the three older age ranges. The performance of a DNN-based model has been already evaluated in another work^35^ for different age-sex-specific groups of patients. It provides better performance compared to our AI model. However, it is important to highlight that there is a difference of 13 years in the mean ages of the AF and SR patients included in the testing cohort of the overall experiment, which considers data from all the different age-sex-specific groups of patients (i.e., mean and std ages are 72.3 $\pm$ 12.7 and 59.0 $\pm$ 16.6 for AF and SR patients, respectively). In their experiments considering age-sex-specific groups (18–49, 50–64, > 65), the authors do not provide information about the mean and standard deviation of the patients in the AF and SR group, therefore it is not possible to know whether both groups are equally distributed or if it happens like in their overall experiment. This can dramatically change the real performance of the AI model as indicated previously in the first phase and Table 2. In our experiment, we minimize such difference by balancing the age distributions of AF and SR patients in each age-sex-specific group of patients and consider thinner age ranges for older patients, subject to a rapid increase of AF incidence.

**Table 4:** Comparison of the performances obtained for age-sex-specific groups of patients with the ResNet model.

	AUC	Sensitivity	Specificity	DOR	F_1_ score
[18, 60)—M	0.61 (0.49–0.72)	33.33% (17.29–52.81)	80.67% (73.43–86.65)	2.09 (0.88–4.93)	0.29 (0.15–0.43)
[60, 70)—M	0.63 (0.52–0.75)	46.67% (28.34–65.67)	71.33% (63.39–78.41)	2.18 (0.98–4.84)	0.32 (0.19–0.44)
[70, 80)—M	0.78 (0.68–0.89)	70.00% (50.60–85.27)	68.00% (59.90–75.37)	4.96 (2.11–11.63)	0.42 (0.30–0.54)
[80, 90)—M	0.76 (0.66–0.87)	83.33% (65.28–94.36)	52.67% (44.36–60.87)	5.56 (2.02–15.31)	0.40 (0.29–0.50)
90 +—M	0.83 (0.72–0.93)	96.67% (82.78–99.92)	46.88% (29.09–65.26)	25.59 (3.10–211.26)	0.76 (0.65–0.86)
[18, 60)—F	0.65 (0.54–0.76)	30.00% (14.73–49.40)	88.67% (82.48–93.26)	3.35 (1.32–8.50)	0.32 (0.15–0.47)
[60, 70)—F	0.73 (0.62–0.84)	36.67% (19.93–56.14)	90.67% (84.84–94.80)	5.62 (2.23–14.17)	0.40 (0.23–0.56)
[70, 80)—F	0.69 (0.58–0.81)	43.33% (25.46–62.57)	79.33% (71.97–85.51)	2.94 (1.29–6.69)	0.35 (0.21–0.49)
[80, 90)—F	0.72 (0.61–0.83)	63.33% (43.86–80.07)	68.67% (60.59–75.98)	3.79 (1.67–8.58)	0.40 (0.27–0.51)
90 +—F	0.76 (0.66–0.87)	80.00% (61.43–92.29)	52.89% (43.61–62.03)	4.49 (1.71–11.78)	0.43 (0.31–0.54)

We evaluate the same ResNet model with ten age-sex-specific test sets. The model is trained with ECG samples that fairly represent all the ten groups of patients. To compute the metrics additional to AUC, we set a unique threshold for the ResNet model, as described in “Methods”. Performances are assessed according to the aggregation rule by average. 95% confidence intervals are reported in brackets.

*M* Male, *F* Female, *AUC* Area Under the Curve, *DOC* Diagnostic Odd Ratio.

### Feature visualization

To conclude the analysis and improve the explainability of the AI models, we apply an algorithm for visualizing the features automatically learned by ResNet model: the Gradient-weighted Class Activation Mapping (Grad-CAM) algorithm, which is usually applied to images to highlights the class-specific discriminative regions of them^36^. Since the architecture of ResNet contains a global average pooling layer followed by a final fully connected layer, we can apply the simplified version of the algorithm, Class Activation Mapping (CAM)^37^, in a straightforward way.Indeed, the global average pooling layer of the ResNet model receives as input two-dimensional matrices from each feature channel, as in the case of images. They represent the temporal sequence of the 12-lead processed signals. The Grad-CAM algorithm for feature visualization allows to identify the portions of ECG signals that the ResNet model exploits to predict the future presence or absence of AF. All the 12 leads present different patterns. We provide examples of the outputs obtained by applying the Grad-CAM algorithm to our ECG signals (Supplementary Figures S2-S5). The importance of the different portions of ECG signals is quantified by numerical values normalized in the interval [0, 1]. Grad-CAM has also been applied in other studies involving ECG signals to visualize the discriminant features learned by DNN-based models^38,39^.

**Figure 1:**
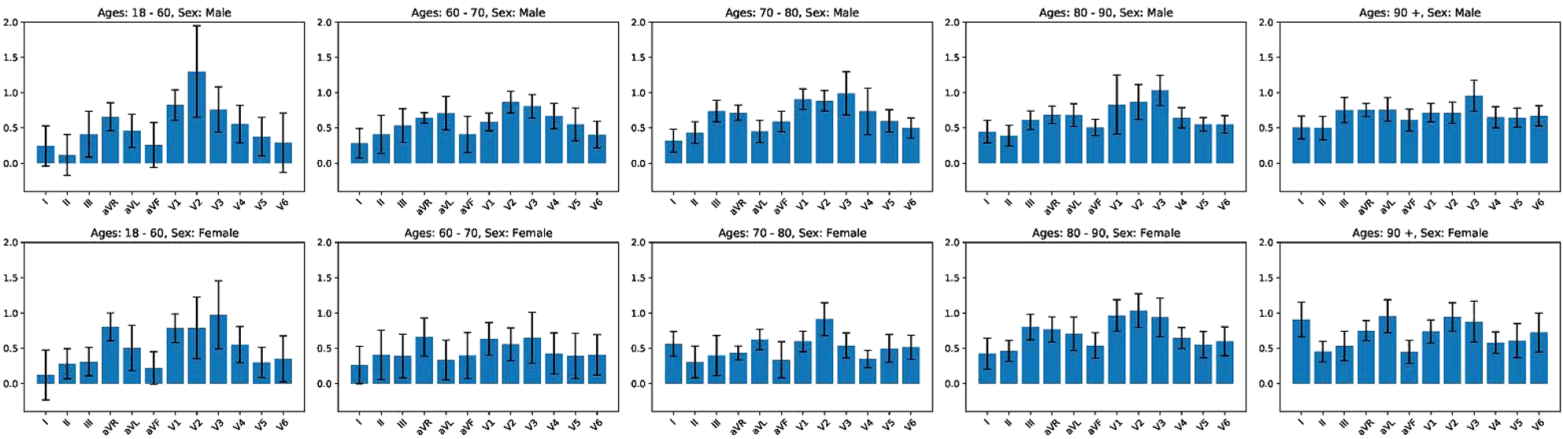
Lead importance in the prediction of future AF. The average contribution of each lead-specific processed signal for the prediction of future AF is shown for the ten different age-sex-specific groups of patients. 95% CIs are also reported for the average values computed.

**Figure 2:**
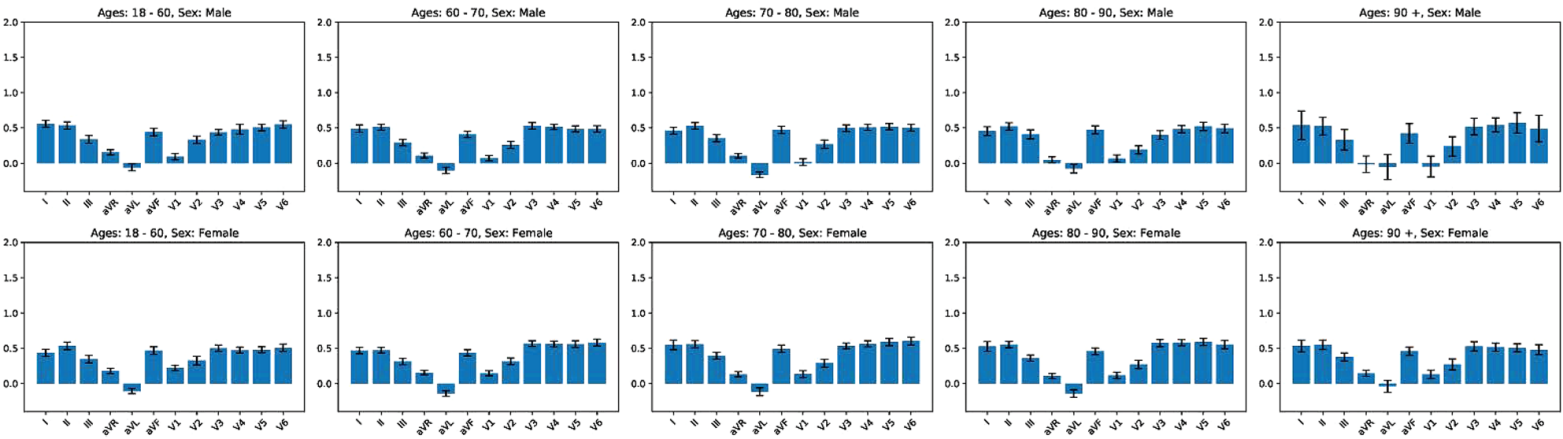
Lead importance in the prediction of AF absence. The average contribution of each lead-specific processed signal for the prediction of AF absence is shown for the ten different age-sex-specific groups of patients. 95% CIs are also reported for the average values computed.

By focusing on the lead-specific values obtained with the Grad-CAM algorithm for each ECG signal, we calculate the average contribution provided by each lead in the prediction of future AF episodes. To examine the correct behavior of our model, we include in this lead-specific analysis only the correctly classified signals of each test set. We observe the differences in the contribution provided by each lead across the different age-sex-specific groups of patients. For the prediction of future AF episodes, all the leads provide a positive contribution in all the age-sex-specific groups of patients. From youth to older patient groups, the contribution provided by each lead tends to increase and the difference between the 12 different contributions tends to decrease. In most of the age-sex-specific groups, leads V2 and V3 are among the leads that provide the highest contribution (Fig. 1). For the prediction of AF absence, the contribution of each lead tends to remain constant between the different age-sex-specific groups. Some leads (aVR, aVL, and V1) provide a minimum contribution, negligible and sometimes negative, in most of the age-sex-specific groups (Fig. 2).

We also analyze the outputs provided by the ResNet model using the Grad-CAM algorithm to identify some recurrent patterns for the prediction of AF episodes and AF absence. We observe that the network focuses on P-waves and first half of QRS-complexes to predict AF episodes, while it focuses on the portions of ECG preceding P-waves and entire QRS-complexes to predict AF absence. Peculiar features automatically learnt by our ResNet model are mostly observable in leads I, aVL, V6 when predicting AF episodes (Supplementary Figures S2-S3) and in leads II, V4, V5 when predicting AF absence (Supplementary Figures S4-S5). Hence the features extracted from lead I, the only lead available when ECGs are measured with non-medical wearable devices^40^, are promising. Wearable devices are less precise but give the possibility of recording ECGs anytime and anywhere. From our observations, P-wave is the key component of ECG signals in the task of AF prediction. P-wave is clearly visible in ECG signals related to AF absence, in contrast with ECG signals preceding AF episodes where P-wave is minimum or missing.

## Discussion

This study provides various insights into prediction of future AF with an AI-enhanced analysis of ECGs recorded during sinus rhythm. At first, we compare the performances of state-of-the-art AI models, three of which are convolutional neural networks (CNNs). CNNs have been employed with good results in literature^23–25^ and allow the application of Grad-CAM algorithm to investigate how the input ECG signals are processed by the network^41^. The results achieved with our three CNN-based models are promising and similar to each other, despite the different output layers of the three models. This fact emphasizes the strength of the features extracted with CNNs at the bottom layers. Also, the best AUC achieved in the scenario of random data sampling in terms of age and sex is consistent with the results of previous studies^23,25^. However, in this study we carry out an analysis of the effect of the age distribution difference between AF and SR groups showing that, without age balancing, we can obtain very high values of AUC just by changing the age distributions of patients. The age of patients is a key aspect to take into consideration before applying AI-based models in real settings in order to obtain meaningful results.

The study also confirms that it is easier to predict AF for old and male patients, in accordance with a previous study where age represents the second most important factor for the AF score predictor proposed^9^. However, the trend of AUC in the different groups of female patients is more unsettled. Multiple age-sex-specific models may be deployed with age-sex-specific datasets when a larger initial database with enough data in each age-sex-specific group is available.

The analysis of lead contribution shows that lead-specific signals are exploited in different ways across age-sex-specific groups of patients. Hence, we expect that age-sex-specific models can extract more accurate age-sex-specific features and achieve better performances compared to a single model.

For the first time, ECG features extracted for AF prediction by an AI-enhanced model are analyzed with a particular focus on lead-specific contributions and age-sex-specific groups of patients. The outcome resulting from the application of Grad-CAM algorithm to our ECG samples can be examined by doctors to acknowledge the behavior of the network and possibly identify subtle traits of ECGs important for AF prediction. The analysis of lead importance across age-sex-specific groups of patients and the comparison of behavior when predicting the presence or absence of AF complement the outcome provided by Grad-CAM.

Our AI-based systems provide promising results and are simple to implement, although it is necessary to overcome some limitations before employing them in real situations. Detailed information about health state, impact of pharmacological and other therapy, and treatments of patients before their first AF onset is fundamental to rule out from the analysis those data with potentially misleading effects. Also, the SR group may include patients with undetected or asymptomatic episodes of paroxysmal AF within the temporal window of recorded ECG. This limitation is common to all the other similar studies. Finally, the availability of additional AF data would reduce the width of CIs in our experiments.

For future work, we can assess the feasibility of training autoencoders with the ECG samples discarded during data cleaning. CNNs can extract robust features from ECGs and an appropriate use of autoencoders would leave more ECGs available for fine-tuning and testing the systems. The outcome provided by Grad-CAM can be further investigated to understand the causes of false predictions, in both AF and SR groups.

## Methods

### Study design and participants

This retrospective study is conducted with a database of ECGs collected from a large cohort of 122,394 patients at La Princesa University Hospital (Madrid, Spain) between May 5, 2010 and February 4, 2019. The Clinical Ethics Committee from Hospital La Princesa approved this study with a waiver of obtaining informed consent from patients. All methods were performed in accordance with the relevant guidelines and regulations. The initial database contains 296,022 12-lead ECGs from 122,394 patients. Each ECG is measured with a sample frequency of 500 Hz and lasts ten seconds. ECGs have been processed by the Philips 12-Lead Algorithm (https://philips.to/36CPabZ) that provides an automatic interpretation of rhythm and extracts discrete variables related to global and lead-specific attributes of the signal. Variables and raw signals are stored in XML files together with information related to patients. Signals are preprocessed to maintain frequencies between 0.7 and 90 Hz and remove frequencies around 50 Hz, noisy due to power supply. We exclude from the study patients with no information about age and patients with only one recorded ECG, since they are not helpful in our analysis.

We identify in the database patients who developed AF at least once, according to the automatic interpretation given by the recording machine. Atrial flutter is treated equally to atrial fibrillation because of their close interrelationship^42^. For each patient we define the AF index date as the date in which the first episode of AF occurred. The automatic interpretation of the ECG rhythm provided by commercial-off-the-shelf (COTS) recording machines is prone to errors: a study showed that the positive predictive accuracy for non-sinus rhythms is only 53.5%^10^. In particular, the automatic interpretation of AF is incorrect between 9.3% and 19% of ECGs^43,44^. On the other hand, the automatic interpretation of sinus rhythm has a positive predictive accuracy of 95%^10^. Hence, we trained a DNN-based classifier of ECG rhythms in order to doubly confirm any interpretation of AF provided by the Philips recording machine. To train and evaluate our rhythm classifier, we used a subset of ECGs manually labelled by cardiologists at La Princesa University Hospital. We achieved AUC of 0.9986 (95% CI 0.9931–1.000) with a test set of 190 ECG samples that equally represented the two rhythms. With the rhythm classifier we computed a score for all those ECGs that represent the first episode of AF for a patient. We discarded patients whose ECG scores are below a fixed threshold of 0.3. In the set of ECGs with the automatic interpretation of AF that we checked, 3,066 of 11,707 ECGs (26.19%) were discarded. This operation aims to minimize the errors due to the interpretation of AF provided by the Philips recording machine, without having to manually check thousands of ECGs.

After this, we divide patients into two groups: (i) the ones who developed AF at least once along their clinical history, and (ii) the ones who only have ECGs in sinus rhythm along their clinical history. For patients in both groups, we only consider ECGs that present an automatic interpretation of “sinus rhythm”, according to our goal of predicting AF from normal-rhythm ECGs that do not contain clear evidence of AF yet. For each patient in the SR group, we define the SR index date as the date of their last recorded ECG. In both groups we apply the following exclusion criteria: ECGs with age < 18 years, with extrasystoles, with atrial to ventricular ratio > 2 or < 1/2, with an average number of P waves per QRS complex ≠ 1, and with number of QRS complexes in the rhythm group higher than the average number of P waves per QRS complex. These rules are set to avoid atrial oversensing secondary to artifacts and to increase the specificity of sinus rhythm diagnosis^9^.

### Time windows

Time windows are defined for each patient according to their AF or SR index date and consist of temporal intervals containing the ECGs of interest in our experiments. Several concerns have been raised regarding ECG selection in^23^: in particular, only the first ECG in a 24-year period is considered for patients in SR group^45^ and the median distance between SR ECGs in the AF group and their AF index date is 0 days^46^. In our study, ECGs in the initial database are collected in a 9-year period and multiple ECGs from the same patient may be selected in experiments, potentially reducing the time distance between ECGs in the AF and SR groups due to the different criteria used for ECG selection. Unless otherwise specified, for each patient in AF group, we consider time windows that include ECGs recorded between two months and one week before their AF index date for the evaluation of the models, and ECGs recorded before their AF index date for the training of models. In this way we avoid the otherwise likely inclusion of SR ECGs recorded after electrical, pharmacological, or spontaneous cardioversion that could alter the ECG in ways detected by the AI algorithm^46^. Differently from other studies^23,25^ and according to a previous work^9^, ECGs recorded in the last week before AF are ruled out for evaluation. Hence, we do not consider ECGs that may refer to hospitalized patients, whose AF may be induced by causes that are out of the scope of this work. Unless otherwise specified, for each patient in SR group we consider only ECGs recorded at least two years before the SR index date. This constraint increases the confidence that SR data employed in the experiments are at least two years far from potential episodes of AF, that may not be included in the initial database.

### AI models

We define six AI models and investigate their ability to predict AF exploiting different kinds of features automatically learned from 12-lead raw ECGs. The first three models have the same bottom layers, obtained from a CNN named Residual Network adapted to time series^41^, and different output layers. The bottom layers consist in three residual blocks, each of them followed by a dropout layer that prevents overfitting. With the same purpose, a gaussian noise with standard deviation equal to 0.1 is added to the processed signal after each dropout layer. All the convolutional layers in the first block have 128 filters, while in the next two blocks they have 256 filters. Within residual blocks, each convolutional layer processes signals along the temporal dimension. Signals are combined along the lead dimension only at last layers.

The last three models are: recurrent neural network (RNN), wavelet scattering, and multivariate logistic regressor (MLR). The input signal of the first four models is two-dimensional: the one-dimensional 10-s signals provided by the different leads are aligned and joined in a second dimension. These models extract features related to the temporal evolution of the ECG signals. For the fifth model, the 12 one-dimensional signals related to single leads are processed individually and combined at a later layer of the architecture. The model extracts features related to the frequency domain of ECG signals. Finally, the input of the sixth model is composed of discrete features extracted from the ECG signals during recording.

The output of each model represents the risk of future AF for patients whose ECGs are provided in input. We call the risk “AF score”. Here we further describe the six models:

- ResNet. After the third residual block, a global average pooling layer averages the two-dimensional values computed for each of the 256 feature channels. The resulting values are processed by a fully connected layer that outputs the AF score.
- ResNet with age and sex inputs. After the third residual block, a global average pooling layer averages the two-dimensional values computed for each of the 256 feature channels. The resulting values are processed by a fully connected layer that outputs four aggregated features. They are concatenated with two numerical values, representing the age and the sex of the patient, and processed by another fully connected layer that outputs the final AF score.
- ResNet + recurrent layers. After the third residual block, a convolutional layer merges values along the lead dimension and outputs a two-dimensional signal, with temporal and filter dimensions, that is processed by two stacked LSTM layers and one fully connected layer to output the AF score.
- Recurrent neural network. In this model, only the first 5 s of ECG signals are processed. They provide results comparable to 10 s-signals at lower computational costs. Max pooling (pool size = 4) is performed on the temporal dimension of each lead-specific signal. Then, the resulting values are processed by two stacked LSTM layers, a dropout layer and a fully connected layer that outputs the AF score.
- Wavelet scattering. Each single-lead signal of the ECG is processed by a wavelet scattering network with an invariance scale equal to 5 s and two filter banks, the first with eight wavelet filters per octave and the second with two filters per octave. The network extracts frequency-related features from the input signal and generates a two-dimensional scattering transform, whose dimensions represent the consecutive time intervals of the signal and the coefficients computed for each of them. For every ECG and time interval, we select the scattering coefficients related to all the 12 leads and provide them as input to another neural network, composed of multiple fully connected layers. The last layer outputs the AF score. For every ECG, the average of AF scores related to the different time intervals is computed.
- Multivariate logistic regressor. This model exploits a subset of the discrete features computed from ECGs during recording. The original model is proposed in another study^9^, where features are selected with statistical analysis. Here we do not consider the 'distance' feature because it assumes two different meanings in the two studies.

### Statistical analysis

Our first five AI models are trained for multiple epochs, until the early stopping condition is fulfilled. They are evaluated on the validation dataset and in the training phase we set not having a decreasing loss for six epochs. The threshold that we consider for the analysis of model accuracy is selected by computing the receiving operation characteristic (ROC) curve of the validation set at the best epoch. At this threshold, the values of sensitivity and specificity are equal. The AI models are implemented in Python version 3 and machine learning packages include scikit-learn and keras. The wavelet scattering network is implemented in MatLab. In the first phase of our analysis, we compute AUC to compare different models and different impacts on performance provided by datasets built in different scenarios. In the second phase, we compute AUC, sensitivity, specificity, diagnostic odd ratio (DOR), and F_1_ score with confidence intervals (CIs) of 95% to accurately evaluate the model performance achieved in different age-sex-specific groups of patients. CIs for AUC and DOR are computed with closed-form expressions, CIs for sensitivity and specificity are computed with MedCalc Software Ltd (https://www.medcalc.org/calc/diagnostic_test.php), and CIs for F_1_ score are computed using bootstrap with 10 000 replications.

## Supplementary Information

Supplementary Information.

## Data Availability

Anonymized data analyzed during the current study are available from the corresponding author on reasonable request, if approved by the Clinical Ethics Committee of the Hospital.
