# Publisher Correction: Analyzing artificial intelligence systems for the prediction of atrial fibrillation from sinus-rhythm ECGs including demographics and feature visualization

**DOI:** 10.1038/s41598-021-03535-x

**Published:** 2021-12-09

**Authors:** Pietro Melzi, Ruben Tolosana, Alberto Cecconi, Ancor Sanz-Garcia, Guillermo J. Ortega, Luis Jesus Jimenez-Borreguero, Ruben Vera-Rodriguez

**Affiliations:** 1 Biometrics and Data Pattern Analytics Lab, Escuela Politecnica Superior, Universidad Autonoma de Madrid, Calle Francisco Tomas Y Valiente, 11, C-235, 28049 Madrid, Spain; 2 Instituto de Investigacion Sanitaria del Hospital Universitario de La Princesa, Madrid, Spain; 3 Science and Technology Department, National University of Quilmes, Bernal, Argentina; 4 Consejo Nacional de Investigaciones Cientificas y Tecnicas, CONICET, Buenos Aires, Argentina; 5 CIBERCV, Centro de Investigacion Biomedica en Red Enfermedades Cardiovasculares, Madrid, Spain

Correction to: *Scientific Reports* 10.1038/s41598-021-02179-1, published online 23 November 2021

The original version of this Article omitted an affiliation for Guillermo J. Ortega. The correct affiliations are listed below.

Instituto de Investigacion Sanitaria del Hospital Universitario de La Princesa, Madrid, Spain

Science and Technology Department, National University of Quilmes, Bernal, Argentina

Consejo Nacional de Investigaciones Cientificas y Tecnicas, CONICET, Buenos Aires, Argentina

The original Article has been corrected.

