# Supplementary material for: Analyzing artificial intelligence systems for the prediction of atrial fibrillation from sinus-rhythm ECGs including demographics and feature visualization
Source: Sci Rep. 2021 Nov 23;11:22786. doi: 10.1038/s41598-021-02179-1 (PMC8610971; doi:10.1038/s41598-021-02179-1)
Supplement: Supplementary file 1 — Supplementary Information. [file 41598_2021_2179_MOESM1_ESM.pdf]

Supplementary Information

Analyzing artificial intelligence systems for the prediction of atrial fibrillation from sinus-rhythm ECGs including demographics and feature visualization

Pietro Melzi, Ruben Tolosana, Alberto Cecconi, Ancor Sanz-Garcia, Guillermo J Ortega, Luis Jesus Jimenez-Borreguero, Ruben Vera-Rodriguez

	**Median age – AF group**	**Median age – SR group**
Training	73 (63-82)	72 (62-81)
Validation	75 (62.8-86.5)	72 (62-86)
Test 18 M	56.75 (48.3-58)	56.125 (48-58)
Test 60 M	63.5 (61.3-67)	63.7 (61.5-67)
Test 70 M	75.1 (71.3-77.4)	74.8 (72-77.4)
Test 80 M	84 (82.1-86)	83.9 (82-86)
Test 90 M	92 (91-94.5)	92 (90.6-93)
Test 18 F	55.75 (51-58)	55.667 (51-58)
Test 60 F	66 (63.3-68)	66 (63.5-68)
Test 70 F	77.333 (74.2-79)	77 (74-78)
Test 80 F	85.367 (83-86.9)	85 (83-87)
Test 90 F	93 (91-95)	92 (91-93)

Supplementary Table S1. Median age of the AF and SR groups data contained in the datasets employed for the analysis of age-sex-specific groups of patients (phase 2). The first and third quartiles are reported in brackets.

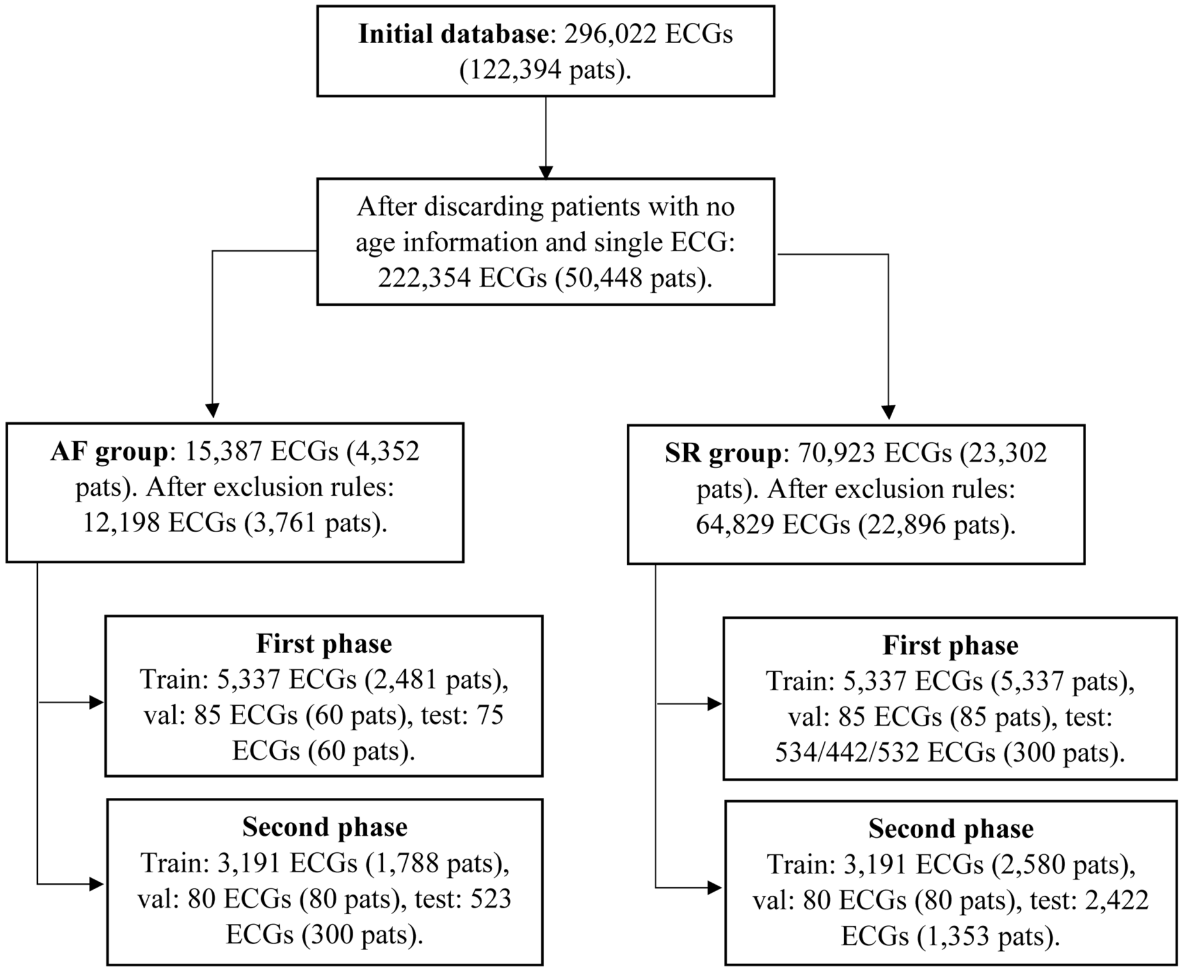

Supplementary Figure S1. **Diagram flow of patients.** Data cleaning and creation of experimental datasets. In the first phase of the experiment, the number of test ECGs for the SR group changes according to the three considered scenarios, but the number of patients does not. In the second phase of the experiment, the number of test patients in the AF group is higher compared to the first experiment because ECGs from multiple age-sex-specific groups of patients need to be tested. AF=Atrial Fibrillation, ECG=Electrocardiogram, SR=Sinus Rhythm.

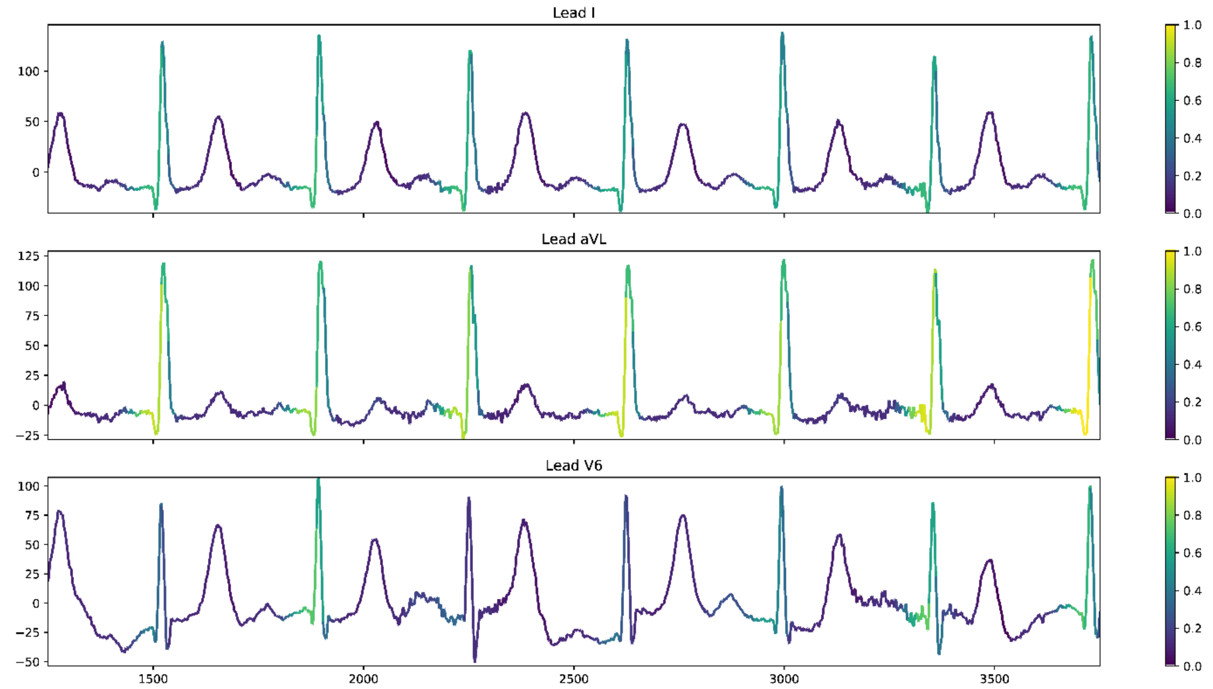

Supplementary Figure S2. **Representative output of Grad-CAM algorithm applied to 12-lead ECG sample in the AF group for the visualization of features that predict future AF.** The different portions of the signal present different colors according to their contribution in the prediction of future AF.

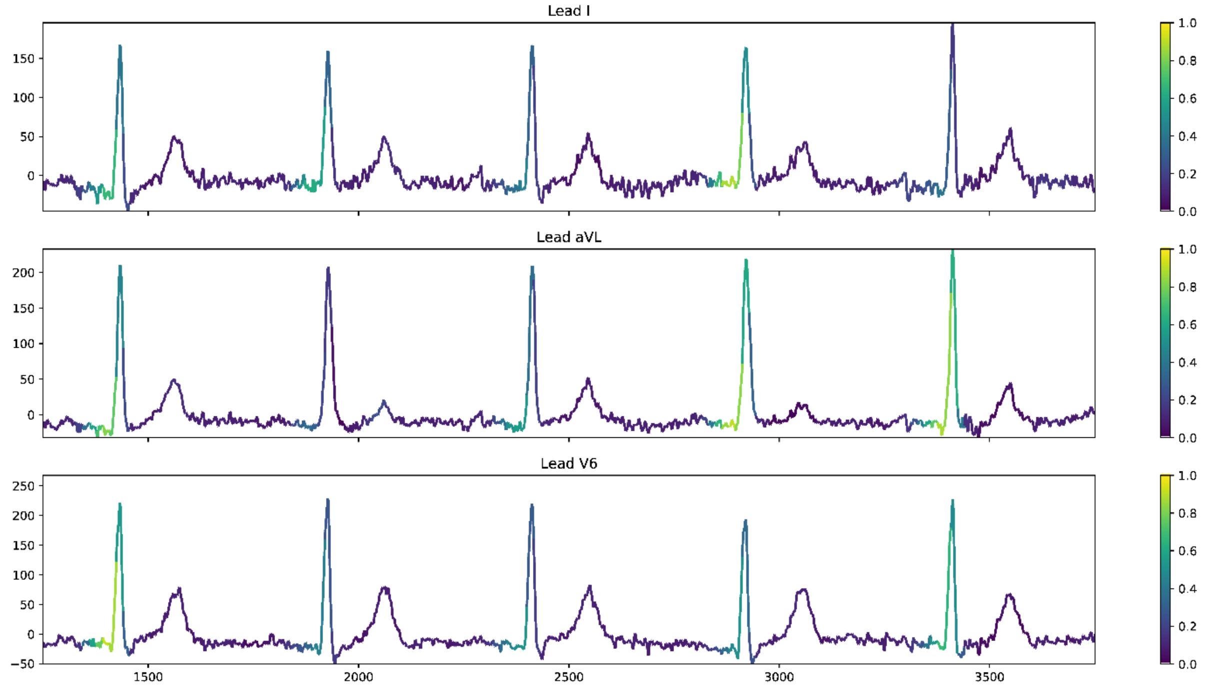

Supplementary Figure S3. **Representative output of Grad-CAM algorithm applied to 12-lead ECG sample in the AF group for the visualization of features that predict future AF.** The different portions of the signal present different colors according to their contribution in the prediction of future AF.

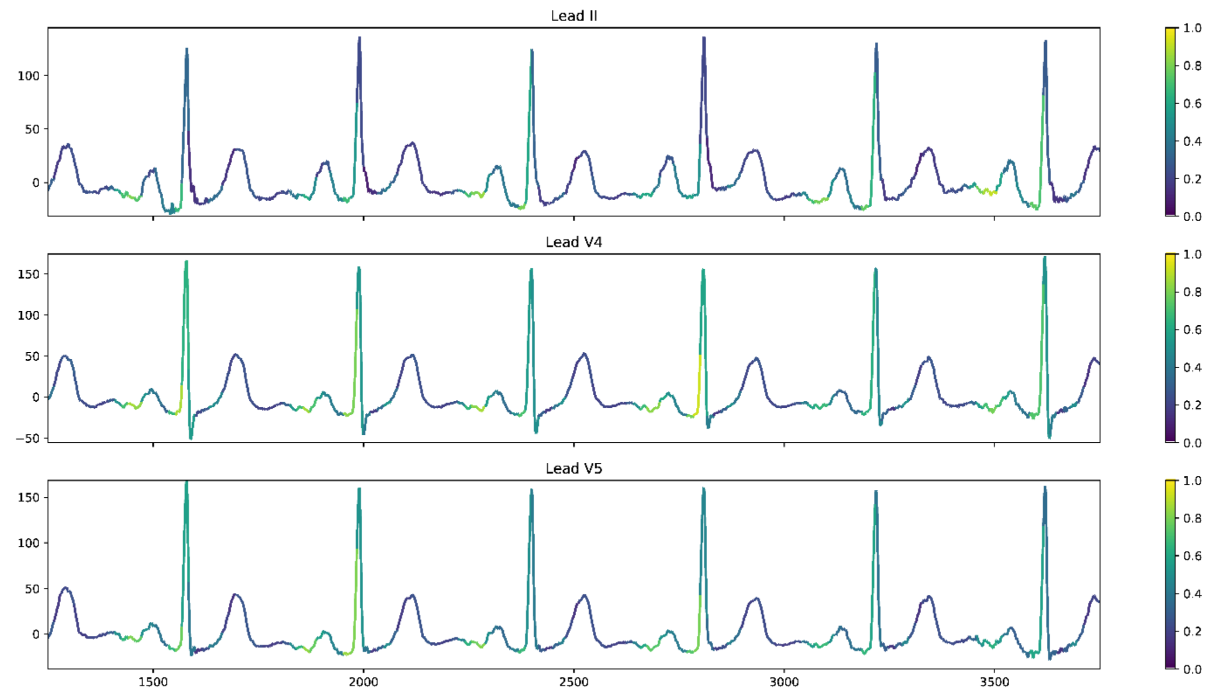

Supplementary Figure S4. **Representative output of Grad-CAM algorithm applied to 12-lead ECG sample in the SR group for the visualization of features that predict AF absence.** The different portions of the signal present different colors according to their contribution in the prediction of AF absence.

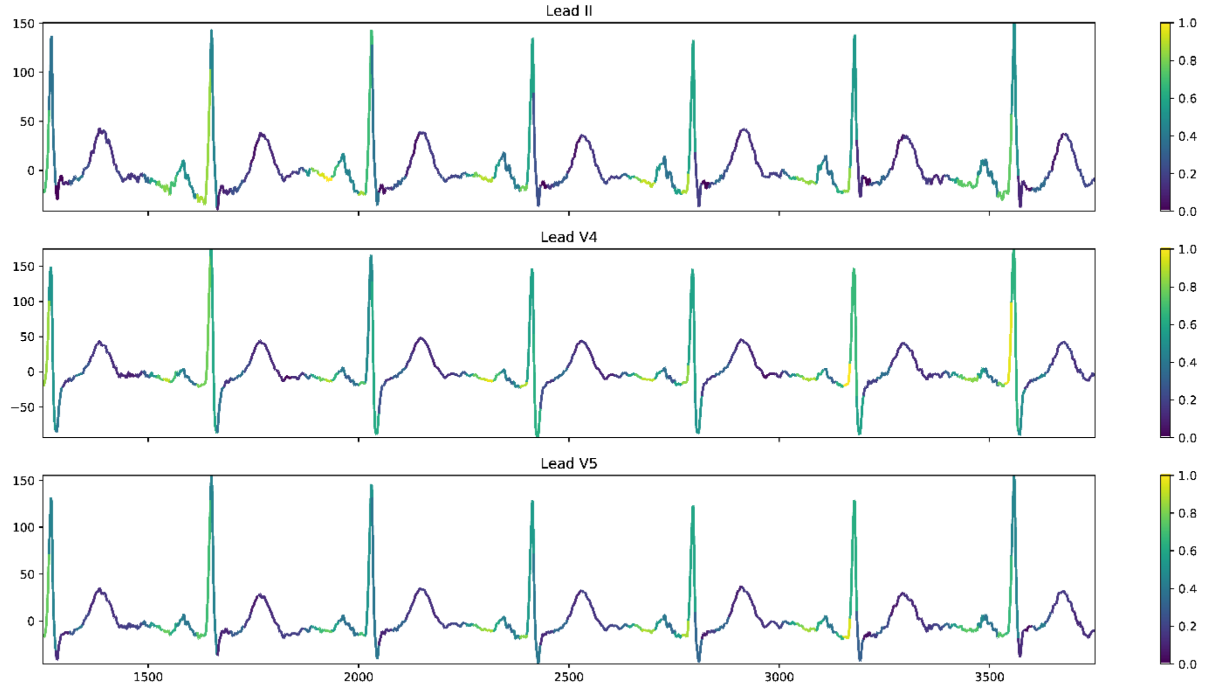

Supplementary Figure S5. **Representative output of Grad-CAM algorithm applied to 12-lead ECG sample in the SR group for the visualization of features that predict AF absence.** The different portions of the signal present different colors according to their contribution in the prediction of AF absence.
